# Supplementary material for: Taxonomic revision of the Malagasy members of the Nesomyrmex angulatus species group using the automated morphological species delineation protocol NC-PART-clustering
Source: PeerJ. 2016 Mar 10;4:e1796. doi: 10.7717/peerj.1796 (PMC4793320; doi:10.7717/peerj.1796)
Supplement: Table S2 — Hymenoptera-specific terminology of morphological statements used in descriptions, identification key, and diagnoses are mapped to classes in phenotype-relevant ontologies. [file peerj-04-1796-s002.docx]

**Table 2.**

| Abbr. | Label | Class genus differentia definition | Comments | uri |
| --- | --- | --- | --- | --- |
| CL | maximum cephalic length in median view | The [median anatomical line](http://api.hymao.org/projects/32/public/ontology_class/show/10209) that extends between the posterior [margin](http://api.hymao.org/projects/32/public/label/show_via_name/margin) of the [cranium](http://api.hymao.org/projects/32/public/ontology_class/show/1269) and the distal [margin](http://api.hymao.org/projects/32/public/label/show_via_name/margin) of the [clypeus](http://api.hymao.org/projects/32/public/ontology_class/show/516) in frontal view. | The maximum cephalic length in median view is not equivalent to the maximum cephalic size that extends between the posterior cranial margin and the distal clypeal line. The head must be carefully tilted to the position with the true maximum. Excavations of hind vertex and/or clypeus reduce CL (Fig. 1A). | <http://purl.obolibrary.org/obo/HAO_0002331> |
| CW | head width | The [anatomical line](http://api.hymao.org/projects/32/public/ontology_class/show/4066) that is the longest horizontal [diameter](http://api.hymao.org/projects/32/public/ontology_class/show/11208) of the [cranium](http://api.hymao.org/projects/32/public/ontology_class/show/1269) in frontal view. | The head width is the largest distance between the lateral margins of the compound eyes measured in frontal view (Fig. 1A). | <http://purl.obolibrary.org/obo/HAO_0002268> |
| CWb | dorsal head width | The [anatomical line](http://api.hymao.org/projects/32/public/ontology_class/show/4066) between the intersections of the [cranium](http://api.hymao.org/projects/32/public/ontology_class/show/1269) contour line and [dorsal head line](http://api.hymao.org/projects/32/public/ontology_class/show/11188) in frontal view. | The dorsal head width is the maximum width of head capsule without the compound eyes that is measured just posterior of the eyes in frontal view (Fig. 1A). | <http://purl.obolibrary.org/obo/HAO_0002314> |
| Cdep | median clypeal notch depth | The [anatomical line](http://api.hymao.org/projects/32/public/ontology_class/show/4066) that is between the [distal clypeal line](http://api.hymao.org/projects/32/public/ontology_class/show/11206) and the proximalmost point of the distal clypeal [notch](http://api.hymao.org/projects/32/public/ontology_class/show/4590) in frontal view. |  | <http://purl.obolibrary.org/obo/HAO_0002333> |
| EL | maximum diameter of compound eye | The longest [diameter](http://portal.hymao.org/projects/32/public/ontology_class/show/11208) of the [eye](http://portal.hymao.org/projects/32/public/ontology_class/show/470). |  | <http://purl.obolibrary.org/obo/HAO_0002326> |
| FRS | frontal carina line | The [transverse torular line](http://portal.hymao.org/projects/32/public/ontology_class/show/11214) that extends between the [frontal carina](http://purl.obolibrary.org/obo/HAO_0001533)e. | Distance of the frontal carinae immediately caudal of the posterior intersection points between frontal carinae and the torular lamellae. If these dorsal lamellae do not laterally surpass the frontal carinae, the deepest point of scape corner pits may be taken as reference line. These pits take up the inner corner of scape base when the scape is fully switched caudad and produce a dark triangular shadow in the lateral frontal lobes immediately posterior of the dorsal lamellae of scape joint capsule (Fig. 1B). | <http://purl.obolibrary.org/obo/HAO_0002323> |
| ML | Weber length | The [anatomical line](http://portal.hymao.org/projects/32/public/ontology_class/show/4066) that connects the global minima of the contour [line](http://portal.hymao.org/projects/32/public/ontology_class/show/1079) of the pronotal slope in [lateral](http://portal.hymao.org/projects/32/public/ontology_class/show/7261) view when the specimen is rotated until the contour [line](http://portal.hymao.org/projects/32/public/ontology_class/show/1079) becames as symmetric as possible and the posteriormost point of the [propodeal lobe](http://portal.hymao.org/projects/32/public/ontology_class/show/11176). | Preferentially measured in lateral view; if the transition point is not well defined, use dorsal view and take the centre of the dark-shaded borderline between pronotal slope and pronotal shield as anterior reference point. In gynes: length from distalmost point of propodeal lobe to the most distant point of steep anterior pronotal face (Fig. 1E). | <http://purl.obolibrary.org/obo/HAO_0002309> |
| MPST | maximum spiracle distance of propodeum | The [anatomical line](http://portal.hymao.org/projects/32/public/ontology_class/show/4066) that connects the center of the [propodeal spiracle](http://portal.hymao.org/projects/32/public/ontology_class/show/4541) with the posteriormost point of the [propodeal lobe](http://portal.hymao.org/projects/32/public/ontology_class/show/11176) in [lateral](http://portal.hymao.org/projects/32/public/ontology_class/show/7261) view. | Maximum distance from the center of the propodeal stigma to the anterioventral corner of the ventrolateral margin of the metapleuron (Fig. 1F). | <http://purl.obolibrary.org/obo/HAO_0002334> |
| MW | mesosoma width | The longest [width](http://portal.hymao.org/projects/32/public/ontology_class/show/11211) of the [pronotum](http://portal.hymao.org/projects/32/public/ontology_class/show/489) in [dorsal](http://portal.hymao.org/projects/32/public/ontology_class/show/4001) view. | Mesosoma width. In workers MW is defined as the longest width of the pronotum in dorsal view excluding the pronotal spines (Fig. 1D). | <http://purl.obolibrary.org/obo/HAO_0002335> |
| NOL | length of petiolar node | The [anatomical line](http://portal.hymao.org/projects/32/public/ontology_class/show/4066) that is the shortest between the center of the petiolar [spiracle](http://portal.hymao.org/projects/32/public/ontology_class/show/590) and the posterior [margin](http://portal.hymao.org/projects/32/public/label/show_via_name/margin) of the petiole in [lateral](http://portal.hymao.org/projects/32/public/ontology_class/show/7261) view. | Length of the petiolar node. Measured in lateral view from the centre of petiolar spiracle to posterodorsal corner of caudal cylinder. Do not erroneously take as reference point the dorso-caudal corner of the helcium, which is sometimes visible (Fig. 1F). | <http://purl.obolibrary.org/obo/HAO_0002336> |
| NOH | maximum height of petiolar node | The [anatomical line](http://api.hymao.org/projects/32/public/ontology_class/show/4066) that is the longest between the [dorsal](http://api.hymao.org/projects/32/public/ontology_class/show/4001) [margin](http://api.hymao.org/projects/32/public/label/show_via_name/margin) of the petiole and the [posterior petiolar distance](http://api.hymao.org/projects/32/public/ontology_class/show/11182) and perpendicular to the [posterior petiolar distance](http://api.hymao.org/projects/32/public/ontology_class/show/11182). |  | <http://purl.obolibrary.org/obo/HAO_0002327> |
| NSTI | apical petiolar spine distance | The [anatomical line](http://api.hymao.org/projects/32/public/ontology_class/show/4066) between the distal ends of the [anterodorsal spines of the petiolar node](http://purl.obolibrary.org/obo/HAO_0002337). | If spine tips are rounded or thick take the centers of spine tips as reference points (Fig. 1C). | <http://purl.obolibrary.org/obo/HAO_0002338> |
| PEH | maximum petiole height | The [anatomical line](http://api.hymao.org/projects/32/public/ontology_class/show/4066) that is the longest between the ventral [margin](http://api.hymao.org/projects/32/public/label/show_via_name/margin) of the petiole and the [dorsal](http://api.hymao.org/projects/32/public/ontology_class/show/4001) [margin](http://api.hymao.org/projects/32/public/label/show_via_name/margin) of the petiole and is perpendicular to the ventral [margin](http://api.hymao.org/projects/32/public/label/show_via_name/margin) of the petiole in [lateral](http://api.hymao.org/projects/32/public/ontology_class/show/7261) view. |  | <http://purl.obolibrary.org/obo/HAO_0002328> |
| PEL | diagonal petiolar length | The [anatomical line](http://api.hymao.org/projects/32/public/ontology_class/show/4066) that extends between the distalmost point of the [subpetiolar process](http://api.hymao.org/projects/32/public/ontology_class/show/6167) and the global minima of the contour [line](http://api.hymao.org/projects/32/public/ontology_class/show/1079) of the [dorsal](http://api.hymao.org/projects/32/public/ontology_class/show/4001) [region](http://api.hymao.org/projects/32/public/ontology_class/show/1078) of the [posterior petiolar constriction](http://api.hymao.org/projects/32/public/ontology_class/show/11181) in [lateral](http://api.hymao.org/projects/32/public/ontology_class/show/7261) view when the specimen is rotated until the contour [line](http://api.hymao.org/projects/32/public/ontology_class/show/1079) became as symmetric as possible. | Fig. 1E. | <http://purl.obolibrary.org/obo/HAO_0002317> |
| PEW | petiole width | The maximum [width](http://api.hymao.org/projects/32/public/ontology_class/show/11211) of the [petiole](http://purl.obolibrary.org/obo/HAO_0000020) in [dorsal](http://api.hymao.org/projects/32/public/ontology_class/show/4001) view. | Anterodorsal spines of the petiolar node are not considered (Fig. 1D). | <http://purl.obolibrary.org/obo/HAO_0002339> |
| PoOC | postocular distance | The [median anatomical line](http://portal.hymao.org/projects/32/public/ontology_class/show/10209) of the [cranium](http://portal.hymao.org/projects/32/public/ontology_class/show/1269) that is the longest between the [dorsal](http://portal.hymao.org/projects/32/public/ontology_class/show/4001) [margin](http://portal.hymao.org/projects/32/public/label/show_via_name/margin) of the [cranium](http://portal.hymao.org/projects/32/public/ontology_class/show/1269) and the [dorsal head width](http://portal.hymao.org/projects/32/public/ontology_class/show/11207). | Use a cross-scaled ocular micrometer and adjust the head to the measuring position of CL. Caudal measuring point: median occipital margin; frontal measuring point: median head at the level of the posterior eye margin (Fig. 1A). | <http://purl.obolibrary.org/obo/HAO_0002340> |
| PPL | postpetiole length | The longest [anatomical line](http://portal.hymao.org/projects/32/public/ontology_class/show/4066) that is perpendicular to the posterior [margin](http://portal.hymao.org/projects/32/public/label/show_via_name/margin) of the [postpetiole](http://portal.hymao.org/projects/32/public/ontology_class/show/4728) in [lateral](http://portal.hymao.org/projects/32/public/ontology_class/show/7261) view and is between the posterior postpetiolar [margin](http://portal.hymao.org/projects/32/public/label/show_via_name/margin) and the anterior postpetiolar [margin](http://portal.hymao.org/projects/32/public/label/show_via_name/margin). | Fig. 1F | <http://purl.obolibrary.org/obo/HAO_0002341> |
| PPW | postpetiole width | The maximum [width](http://portal.hymao.org/projects/32/public/ontology_class/show/11211) of the [postpetiole](http://portal.hymao.org/projects/32/public/ontology_class/show/4728) in [dorsal](http://portal.hymao.org/projects/32/public/ontology_class/show/4001) view. | Fig. 1D | <http://purl.obolibrary.org/obo/HAO_0002342> |
| PSTI | apical distance of pronotal spines | The [anatomical line](http://portal.hymao.org/projects/32/public/ontology_class/show/4066) between the distal ends of the [pronotal spines](http://purl.obolibrary.org/obo/HAO_0002344). | If spine tips are rounded or thick take the centers of spine tips as reference points (Fig. 1D). | <http://purl.obolibrary.org/obo/HAO_0002345> |
| SL | scape length | The [proximodistal anatomical line](http://portal.hymao.org/projects/32/public/ontology_class/show/10210) of the scapal [area](http://portal.hymao.org/projects/32/public/ontology_class/show/1080) distal to the [radicle](http://portal.hymao.org/projects/32/public/ontology_class/show/551). | Maximum straight line scape length excluding the radicle (Fig. 1A). | <http://purl.obolibrary.org/obo/HAO_0002346> |
| SPBA | minimum spine distance | The shortest [anatomical line](http://portal.hymao.org/projects/32/public/ontology_class/show/4066) between the [lateral](http://portal.hymao.org/projects/32/public/ontology_class/show/7261) margins of the [propodeal spine](http://purl.obolibrary.org/obo/HAO_0001695)s. | This should be measured in anterodorsal view, since the wider parts of the ventral propodeum do not interfere with the measurement in this position. If the lateral margins of spines diverge continuously from the tip to the base, a smallest distance at base is not defined. In this case, SPBA is measured at the level of the bottom of the interspinal meniscus (Fig. 1C). | <http://purl.obolibrary.org/obo/HAO_0002347> |
| SPST | spine length | The [anatomical line](http://portal.hymao.org/projects/32/public/ontology_class/show/4066) between the center of the [propodeal spiracle](http://portal.hymao.org/projects/32/public/ontology_class/show/4541) and the distal end of the [propodeal spine](http://portal.hymao.org/projects/32/public/ontology_class/show/6166). | Spine length. Distance between the centre of propodeal stigma and spine tip. The stigma centre refers to the midpoint defined by the outer cuticular ring but not to the centre of real stigma opening that may be positioned eccentrically (Fig. 1F). | <http://purl.obolibrary.org/obo/HAO_0002348> |
| SPTI | apical spine distance | The [anatomical line](http://portal.hymao.org/projects/32/public/ontology_class/show/4066) between the distal ends of the [propodeal spines](http://purl.obolibrary.org/obo/HAO_0001695). | If spine tips are rounded or truncated, the centres of spine tips are taken as reference points (Fig. 1C). | <http://purl.obolibrary.org/obo/HAO_0002319> |
|  | anterior pronotal slope | The concave [area](http://api.hymao.org/projects/32/public/ontology_class/show/1080) anteriorly on the [mesosoma](http://api.hymao.org/projects/32/public/ontology_class/show/473) that accommodates the posterior [area](http://api.hymao.org/projects/32/public/ontology_class/show/1080) of the [cranium](http://api.hymao.org/projects/32/public/ontology_class/show/1269). |  | <http://purl.obolibrary.org/obo/HAO_0002311> |
|  | anterior setal pit | The anteriormost [setal pit](http://api.hymao.org/projects/32/public/ontology_class/show/9370) on the [dorsal](http://api.hymao.org/projects/32/public/ontology_class/show/4001) side of the petiole. |  | <http://purl.obolibrary.org/obo/HAO_0002312> |
|  | caudal cylinder | The petiolar [area](http://api.hymao.org/projects/32/public/ontology_class/show/1080) posterior to the [posterior petiolar constriction](http://api.hymao.org/projects/32/public/ontology_class/show/11181). |  | <http://purl.obolibrary.org/obo/HAO_0002318> |
|  | cranial scrobe of the pronotum | The [scrobe](http://api.hymao.org/projects/32/public/ontology_class/show/588) on the [pronotum](http://api.hymao.org/projects/32/public/ontology_class/show/489) that accommodates the posterior surface of the [cranium](http://api.hymao.org/projects/32/public/ontology_class/show/1269). |  | <http://purl.obolibrary.org/obo/HAO_0002343> |
|  | distal clypeal line | The [anatomical line](http://api.hymao.org/projects/32/public/ontology_class/show/4066) that is perpendicular to the [median anatomical line](http://api.hymao.org/projects/32/public/ontology_class/show/10209) and is the tangent at the distalmost point(s) of the [clypeus](http://api.hymao.org/projects/32/public/ontology_class/show/516) in frontal view. |  | <http://purl.obolibrary.org/obo/HAO_0002316> |
|  | dorsal head line | The [anatomical line](http://api.hymao.org/projects/32/public/ontology_class/show/4066) between the posteriormost (dorsalmost) points of compound eyes in frontal view. |  | <http://purl.obolibrary.org/obo/HAO_0002315> |
|  | dorsal petiolar scrobe | The [scrobe](http://api.hymao.org/projects/32/public/ontology_class/show/588) that is [dorsal](http://api.hymao.org/projects/32/public/ontology_class/show/4001) to the [propodeal foramen](http://api.hymao.org/projects/32/public/ontology_class/show/1074) and accommodates the proximodorsal [area](http://api.hymao.org/projects/32/public/ontology_class/show/1080) of the petiole. |  | <http://purl.obolibrary.org/obo/HAO_0002313> |
|  | external area of the scape | The [area](http://api.hymao.org/projects/32/public/ontology_class/show/1080) of the [scape](http://api.hymao.org/projects/32/public/ontology_class/show/550) that faces away from the cranial surface in fully caudal [scape](http://api.hymao.org/projects/32/public/ontology_class/show/550) position. |  | <http://purl.obolibrary.org/obo/HAO_0002320> |
|  | eye | The [compound organ](http://api.hymao.org/projects/32/public/ontology_class/show/4065) that is composed of ommatidia. |  | <http://purl.obolibrary.org/obo/HAO_0000217> |
|  | facial area of the scape | The [area](http://api.hymao.org/projects/32/public/ontology_class/show/1080) of the [scape](http://api.hymao.org/projects/32/public/ontology_class/show/550) that faces the [cranium](http://api.hymao.org/projects/32/public/ontology_class/show/1269) surface when the [scape](http://api.hymao.org/projects/32/public/ontology_class/show/550) is in fully flexed position. |  | <http://purl.obolibrary.org/obo/HAO_0002321> |
|  | frontal carina | The [carina](http://api.hymao.org/projects/32/public/label/show_via_name/carina) that extends along the [lateral](http://api.hymao.org/projects/32/public/ontology_class/show/7261) [margin](http://api.hymao.org/projects/32/public/label/show_via_name/margin) of the intertorular [area](http://api.hymao.org/projects/32/public/ontology_class/show/1080) (median [margin](http://api.hymao.org/projects/32/public/label/show_via_name/margin) of the [antennal foramen](http://api.hymao.org/projects/32/public/ontology_class/show/611)) towards the [vertex](http://api.hymao.org/projects/32/public/label/show_via_name/vertex). |  | <http://purl.obolibrary.org/obo/HAO_0001533> |
|  | frontal carina line | The [transverse torular line](http://api.hymao.org/projects/32/public/ontology_class/show/11214) that extends between the [frontal carina](http://purl.obolibrary.org/obo/HAO_0001533)e. |  | <http://purl.obolibrary.org/obo/HAO_0002323> |
|  | lateral carina of clypeus | The [carina](http://api.hymao.org/projects/32/public/label/show_via_name/carina) that extends between the ventral (anterior) [margin](http://api.hymao.org/projects/32/public/label/show_via_name/margin) of the [antennal foramen](http://api.hymao.org/projects/32/public/ontology_class/show/611) to the [apical](http://api.hymao.org/projects/32/public/ontology_class/show/5985) clypeal [margin](http://api.hymao.org/projects/32/public/label/show_via_name/margin). |  | <http://purl.obolibrary.org/obo/HAO_0002324> |
|  | margin | The [line](http://api.hymao.org/projects/32/public/ontology_class/show/1079) that delimits the periphery of an [area](http://api.hymao.org/projects/32/public/ontology_class/show/1080). |  | <http://purl.obolibrary.org/obo/HAO_0000510> |
|  | median clypeal notch | The median [notch](http://api.hymao.org/projects/32/public/ontology_class/show/4590) that is on the distal clypeal [margin](http://api.hymao.org/projects/32/public/label/show_via_name/margin). |  | <http://purl.obolibrary.org/obo/HAO_0002332> |
|  | mesosoma | The [anatomical cluster](http://api.hymao.org/projects/32/public/ontology_class/show/3968) that is composed of the [prothorax](http://api.hymao.org/projects/32/public/ontology_class/show/476), [mesothorax](http://api.hymao.org/projects/32/public/ontology_class/show/477) and the [metapectal-propodeal complex](http://api.hymao.org/projects/32/public/ontology_class/show/1248). |  | <http://purl.obolibrary.org/obo/HAO_0000576> |
|  | Weber length | The [anatomical line](http://api.hymao.org/projects/32/public/ontology_class/show/4066) that connects the global minima of the contour [line](http://api.hymao.org/projects/32/public/ontology_class/show/1079) of the pronotal slope in [lateral](http://api.hymao.org/projects/32/public/ontology_class/show/7261) view when the specimen is rotated until the contour [line](http://api.hymao.org/projects/32/public/ontology_class/show/1079) becames as symmetric as possible and the posteriormost point of the [propodeal lobe](http://api.hymao.org/projects/32/public/ontology_class/show/11176). |  | <http://purl.obolibrary.org/obo/HAO_0002309> |
|  | petiolar scrobe | The [scrobe](http://api.hymao.org/projects/32/public/ontology_class/show/588) that is located ventrally of the [propodeal foramen](http://api.hymao.org/projects/32/public/ontology_class/show/1074) and accommodates the [proximal](http://api.hymao.org/projects/32/public/ontology_class/show/7246) [area](http://api.hymao.org/projects/32/public/ontology_class/show/1080) of the petiole. |  | <http://purl.obolibrary.org/obo/HAO_0002265> |
|  | pronotal spine | The [spine](http://api.hymao.org/projects/32/public/ontology_class/show/1143) that is located at the dorsolateral [edge](http://api.hymao.org/projects/32/public/ontology_class/show/4533) of the [cranial scrobe of the pronotum](http://api.hymao.org/projects/32/public/ontology_class/show/11221). |  | <http://purl.obolibrary.org/obo/HAO_0002344> |
|  | pronotum | The [notum](http://api.hymao.org/projects/32/public/ontology_class/show/591) that is located in the [prothorax](http://api.hymao.org/projects/32/public/ontology_class/show/476). |  | <http://purl.obolibrary.org/obo/HAO_0000853> |
|  | scape | The [antennal segment](http://api.hymao.org/projects/32/public/ontology_class/show/4376) that is [proximal](http://api.hymao.org/projects/32/public/ontology_class/show/7246) to the [pedicel](http://api.hymao.org/projects/32/public/ontology_class/show/541) and is connected to the [head](http://api.hymao.org/projects/32/public/ontology_class/show/469) via the [radicle](http://api.hymao.org/projects/32/public/ontology_class/show/551). |  | <http://purl.obolibrary.org/obo/HAO_0000908> |
|  | scrobe | The [area](http://api.hymao.org/projects/32/public/ontology_class/show/1080) that is impressed and is for the reception or concealment of another [sclerite](http://api.hymao.org/projects/32/public/ontology_class/show/584). |  | <http://purl.obolibrary.org/obo/HAO_0000912> |
|  | setal angle | The [angle](http://api.hymao.org/projects/32/public/label/show_via_name/angle) of the proximodistal axis of the [seta](http://api.hymao.org/projects/32/public/ontology_class/show/616) to the contour [line](http://api.hymao.org/projects/32/public/ontology_class/show/1079) of the bodypart where the [seta](http://api.hymao.org/projects/32/public/ontology_class/show/616) is located. |  | <http://purl.obolibrary.org/obo/HAO_0002330> |
|  | setal line | The [row](http://api.hymao.org/projects/32/public/ontology_class/show/3996) that is composed of setae. |  | <http://purl.obolibrary.org/obo/HAO_0000903> |
|  | setal pit | The [impression](http://api.hymao.org/projects/32/public/ontology_class/show/1085) with a centered [sensillum trichodeum](http://api.hymao.org/projects/32/public/ontology_class/show/10425). |  | <http://purl.obolibrary.org/obo/HAO_0001958> |
|  | spine | The [process](http://api.hymao.org/projects/32/public/ontology_class/show/1075) that lacks non-sclerotised ring at the base. |  | <http://purl.obolibrary.org/obo/HAO_0000949> |
|  | spiracle | The [anatomical cluster](http://api.hymao.org/projects/32/public/ontology_class/show/3968) that is composed of the distal end of the trachea and the [margin](http://api.hymao.org/projects/32/public/label/show_via_name/margin) of the [sclerite](http://api.hymao.org/projects/32/public/ontology_class/show/584) or [conjunctiva](http://api.hymao.org/projects/32/public/ontology_class/show/1072) surrounding the spiracular opening. |  | <http://purl.obolibrary.org/obo/HAO_0000950> |
|  | transverse torular line | The [anatomical line](http://api.hymao.org/projects/32/public/ontology_class/show/4066) that is tangential to the posteriormost points of the [antennal rim](http://purl.obolibrary.org/obo/HAO_0000103)s. |  | <http://purl.obolibrary.org/obo/HAO_0002322> |
|  | width | A 1-D extent quality which is equal to the distance from one side of an object to another side which is opposite. |  | <http://purl.obolibrary.org/obo/HAO_0002308> |
