# Supplementary material for: Taxonomic revision of the Malagasy members of the Nesomyrmex angulatus species group using the automated morphological species delineation protocol NC-PART-clustering
Source: PeerJ. 2016 Mar 10;4:e1796. doi: 10.7717/peerj.1796 (PMC4793320; doi:10.7717/peerj.1796)
Supplement: Supplemental Information 1 — R script of NC clustering and method PART implementing cluster methods “hclust” and “kmeans”. Mark dendrogram function mapping the results of partitioning algorithm PART on the dendrogram is also added. [file peerj-04-1796-s004.docx]

rm(list=ls()); ls() # tidying up the work space

library(MASS) # setup environment

library(cluster)

d <- read.csv("~angulatus-group_rawdata.csv", header=TRUE,na.strings="") # loading the input file of the data

vari<-c(4:24) # define columns with (morphometric) variables

grouping<-2 # define grouping variable (column)

spname<-3 # define species name (column)

summary(d)

dim(d); n<-dim(d)[1] # identify dimensions

ddrop<-rep(FALSE,n) # make empty vector ddrop (n = number of measurement variables)

ddrop<-is.na(d[,grouping]); colnames(d)[grouping]

sum(ddrop)

dm<-d[!ddrop,vari]; summary(dm) # dm is data matrix

sum(is.na(dm)) # check for missing values

n<-dim(dm)[1] # number of observations (rows)

gid<-paste(d[!ddrop,spname],"-",as.character(d[!ddrop,grouping]),sep="")

#gid # show (new) sample names

table(gid);length(table(gid));sum(is.na(gid))

ddrop2<-NULL

tmp<-apply(apply(dm,1,is.na),2,sum)>0 # search for zeros

tmp3<-is.na(gid) # check for missing values

ddrop2<- tmp | tmp3;

rm(tmp,tmp3)

sum(ddrop2)

dm<-dm[!ddrop2,]; summary(dm)

sum(is.na(dm)) # check for missing values

n<-dim(dm)[1]

gid<-gid[!ddrop2]

table(gid);length(table(gid));sum(is.na(gid))

#LDA using nest samples as groups

formlda<-lda(dm,as.factor(gid)); formlda # gid=group ID (site)

predlda<-predict(formlda,dm)$class

sum(predlda==gid)/length(gid)

x<-table(predlda==gid,gid); x

mpredlda<-predict(formlda,formlda$means)$x

plot(agnes(mpredlda,method="average"),which.plots=2,hang=-1,cex=0.7)

###########################################################################

### PART Partitioning based on recursive thresholging

###########################################################################

library(clusterGenomics)

groups1 = dm$gid

#Run PART.hclust

res <- part(mpredlda,Kmax=14,minSize=5,Kmax.rec=8,B=1000, cl.method = "hclust",

linkage="average") # run PART with cluster method 'hclust' using default setup

cbind(res$lab.hatK, groups1) #extract hypothesis

summary.hclust<-cbind(res$lab.hatK, groups1)

hyp.part.hclust <- summary.hclust

thyp.part.hclust <- t(hyp.part.hclust) # transpose partitioning results

#Run PART.kmeans

resa <- part(mpredlda, cl.method = "kmeans", minSize=5,Kmax.rec=8,nstart = 10,B=1000)

cbind(resa$lab.hatK, groups1) #extract hypothesis

summary.kmeans<-cbind(resa$lab.hatK, groups1)

hyp.part.kmeans <- summary.kmeans

thyp.part.kmeans <- t(hyp.part.kmeans) # transpose partitioning results

###########################################################################

### Let's map the results of method PART on the NC clustering dendrogram

###########################################################################

#Source of the following fragment of the script: https://r-forge.r-#project.org/scm/viewvc.php/src/hyperSpec/R/mark.dendrogram.R?#root=hyperspec&r1=451&r2=450&pathrev=451&diff_format=s, see R package hyperSpec

dend <- agnes(mpredlda,method="average")

par (xpd = TRUE, mar = c (10, 4, 4, 2)) # allows plotting into the margin

par(mfrow=c(1,1))

plot(dend,which.plots=2,hang=-0.1,cex=0.65) # set letter size

G2 <- c(1:12) # optionally type final species hypothesis, separated by comma

dend$Group <- G2

clusters <- as.factor (dend$Group)

Z<-0.05 # set the bars' height (dendrogram$height in mark.dendrogram function)

mark.dendrogram <- function (dendrogram, groups, col = seq_along (unique (dend$Group)),

pos.marker = pos.marker,

height = Z * max (dendrogram$height),

pos.text = pos.text,

border = NA, text.col = "white", label, label.right = FALSE,

...){

if (! is.factor (groups))

groups <- as.factor (dend$Group)

groups.x <- groups [dendrogram$order] # clusters in order on x axis

rle.groups <- rle (as.integer (groups.x)) # run-length encoding gives borders

end <- cumsum (rle.groups$lengths) + 0.5

start <- c (0.5, (head (end, -1)))

text <- (start + end) / 2

text.col <- rep (text.col, length.out = length (text))

for (g in seq_along (rle.groups$lengths)){

rect (xleft = start [g], ybottom = pos.marker - height,

xright = end [g], ytop = pos.marker,

col = col [rle.groups$values[g]], border = border, ...)

if (! is.na (text.col [g]))

text (x = text [g], y = pos.text,

levels (groups) [rle.groups$values [g]],

col = text.col [rle.groups$values [g]], ...)

}

if (! missing (label))

text (x = label.right * tail (end, 1) * 1.01, y = pos.marker - height/2,

label, adj = c(1 - label.right, .3))

}

mark.dendrogram (dend, as.factor (thyp.part.hclust), pos.marker = -32, pos.text = -33, cex=0.7, label = "'part.hclust'") # display partitioning results of cl.method hclust on the dendrogram

mark.dendrogram (dend, as.factor (thyp.part.kmeans), pos.marker = -36, pos.text = -37, cex=0.7, label = "'part.kmeans'") # display partitioning results of cl.method kmeans on the dendrogram

mark.dendrogram (dend, as.factor (dend$Group), pos.marker = -300, pos.text = -300, cex=0.6, label = "'final.sp.hyp'") # optional: read final species hypothesis
